# Zinc Metalloprotease SlMEP1: An Essential Factor Required for Fungal Virulence in *Stemphylium lycopersici*

**DOI:** 10.3390/jof11050330

**Published:** 2025-04-22

**Authors:** Dezhen Zhang, Wenjuan Chi, Tingting Li, Cuicui Wang, Jing Zhang, Haoqin Pan, Ning Qiao, Jintang Li, Xiaoan Sun

**Affiliations:** 1 Shandong Provincial University Laboratory for Protected Horticulture, Weifang University of Science and Technology, Weifang 262700, China; zhen5198@126.com (D.Z.); rmlt@163.com (T.L.); wangcuicui1111@126.com (C.W.); zhangj10536@wfust.edu.cn (J.Z.); never423@163.com (H.P.); qning78@163.com (N.Q.); li_jintang@163.com (J.L.); 2 Division of Plant Industry, Florida Department of Agriculture and Consumer Services, Gainesville, FL 32607, USA

**Keywords:** *Stemphylium lycopersici*, metalloprotease, pathogenicity, tomato

## Abstract

*Stemphylium lycopersici* is the causal pathogen of the devastating tomato gray leaf spot with a wide range of alternative plant hosts. To mitigate its potential endemic in facility-cultivated tomatoes, novel disease control strategies should be attempted to minimize the use of chemical fungicides. In this study, we identified a metalloprotease from *S. lycopersici* genome and designated it as SlMEP1, as it appears to be a typical zinc metalloproteinase containing a WLM (WSS1-like metalloprotease) domain and a characteristic HEXXH motif, which we determined by analyzing its transcriptional profile and enzymatic functions. The transcription level of *SlMEP1* increased greatly during the fungal invasion of tomato leaves. The deletion of the *SlMEP1* gene from *S. lycopersici* hindered its mycelial growth and reduced its pathogenicity. An assessment of the functional dissection indicated that SlMEP1 induced cell collapse and inhibited the expression of the host chitinases, which consequently made tomato cells more susceptible to *S. lycopersici* and other pathogenic fungi.

## 1. Introduction

Tomato (*Solanum lycopersicum*) gray leaf spot (TGLS) is one of the most significant and potentially devastating diseases of tomatoes, especially of facility-cultivated crops. It poses a threat to tomato production worldwide [1,2]. In China, the disease was first reported in 2002 in Yutai County, Shandong Province, where during the survey, the endemic saw an average of 43% disease incidence in over 30,000 tomato-growing greenhouses and a 90% disease incidence in 2003 [3], resulting in a reduction in fruit quality and a yield and economic loss of approximately 20–80%. *S. lycopersici*, as the major causal agent of the disease, has a wide range of hosts, such as peppers, which were infected in the winter of 2019, resulting in a loss of 50% of the plants in the Beijing production areas [4]. The management of TGLS is a challenge due to lack of host resistance and inadequate knowledge of the pathogen’s pathogenesis. Therefore, understanding the pathogenesis of *S. lycoperisici* becomes imperatively important in order to develop a control strategy and design novel and effective bio-pesticides for disease control.

Metalloproteases (MEPs) belong to multiple families of hydrolytic enzymes that contain metal ions in their active centers [5,6,7]. Among them, zinc MEPs contain zinc ion-binding motifs that are characterized by common HEXXH sequences and belong to the zincin superfamily [8,9,10]. Zinc MEPs are reported to be essential for certain fungal plant pathogens to be virulent [11,12,13,14]. Two zinc MEPs, Mrmep1 and Mrmep2, from the entomopathogenic fungus *Metarhizium robertsii*, are reportedly involved in its growth, cell wall integrity, sporulation, virulence, and pathogenesis [7]. The fungalysin MEP Cgfl of *Colletotrichum graminicola*, which causes maize anthracnose, is found to be strongly upregulated during the early stage of the infection so as to enhance fungal virulence as an effector [14,15]. Regarding *Rhizoctonia cerealis*, the zinc MEP RcMEP1 of the M43 family, MEP RcMEP2 of the M35 family, and MEP RcFL1 of the M36 family were functionally analyzed, respectively, for their involvement in pathogenicity through different mechanisms [16,17,18]. In *Fusarium graminearum*, the MEP FgFly1 of the M36 family is found to regulate the fungal pathogenicity by interacting with the CAMTA protein in host plant cells [10], while the zinc MEP FgM35 of the M35 family plays an indispensable role both in the reproductive process and in the pathogenicity of *F. graminearum* through interacting with the zinc-binding TaZnBP protein in wheat to enhance plant immunity [19]. However, little is known about the functions of MEPs in *S. lycopersici*. Our preliminary work on the RNA-sequencing and the transcript dynamics of genes in *S. lycopersici* strain SL1216, isolated from infected tomatoes indicates that *SlMEP1* encoding a zinc MEP was upregulated during the early fungal infection [20], suggesting that SlMEP1 might have played a role in fungal pathogenicity throughout the whole process of pathogenesis.

Chitinases are pathogenesis-related proteins that contribute to plant defense against fungal infections [21] through inhibiting fungal growth via the hydrolysis of chitin as a main structural component of fungal cell walls [17] and/or through triggering plant immune responses as a microbe-associated molecular pattern [14]. To disrupt the chitin-induced immune system of host plants, some pathogenic fungi produce MEPs during infection [14,22,23,24]. A fungalysin MEP of *F. verticillioides* cleaves within a sequence that is conserved in class IV chitinases [22]. An MEP and serine protease from *F. oxysporum* f. sp. *lycopersici* act synergistically to cleave tomato chitinases and reduce host antifungal activities [24]. To date, no MEP functions of *S. lycopersici* relating to chitinases in tomatoes have been reported.

In this study, we intended to identify a zinc MEP-encoding gene (*SlMEP1*) from the *S. lycopersici* strain SL1216, investigate its expression pattern during the infection process in tomatoes, and characterize the functional role in the fungal pathogenesis. Our results should reveal that *SlMEP1* acts as a pathogenicity-required factor by inducing plant cell collapse and inhibiting the expression of tomato chitinases, which can provide an experimental basis for further studies on the phytopathogenic mechanism and pathogenicity–host interactions.

## 2. Materials and Methods

### 2.1. Preparation of Tomato Plants and Fungal Cultures

Gray leaf spot-susceptible tomato plants (cultivar “Jinpeng No. 1”) were grown in a glasshouse at 25 °C with a 16 h light–8 h dark schedule. A highly virulent strain of *S. lycopersici*, SL1216, isolated from infected tomato plants in Shouguang, Shandong Province, China [20], was cultured on V8 juice agar plates [25] at 25 °C for 5 days. The mycelial disks were cut using a sterile puncher and inoculated onto tomato leaves to induce spore production. Harvested spores were suspended with sterile ddH_2_O and were adjusted to a concentration of 1 × 10^5^ CPU/mL for further use.

### 2.2. Vegetative Growth Assays and Observation of Mycelial Morphology

The wild-type strain SL1216, the mutant strain Δ*SlMEP1*, and the complemented strain Δ*SlMEP1*-C were placed, respectively, onto the PDA, CMA, and V8 agar plates and cultured at 25 °C for 5 days to observe their morphological features and measure their mycelial growth rate. Photographs were taken for their colony and hyphal branching at the edge of V8-coated slides using an Olympus microscope 24 h post cultivation (Olympus, Tokyo, Japan).

### 2.3. Phylogenetic Analysis and Sequence Alignment

Multiple alignments were determined using the DNAMAN 6.0 software (Lynnon Biosoft, San Ramon, CA, USA), and a neighbor-joining (NJ) tree was constructed with 1000 bootstrap replicates using MEGA (version 7.0, MEGA Inc., Memphis, TN, USA) [26] based on the multiple sequence alignments of the zinc MEP encoding sequence of *S. lycopersici* (CIDEFI-216 and SL1216) and 12 zinc MEPs of other fungi downloaded from the NCBI database. A homology alignment (http://pfam.xfam.org/, accessed on 10 February 2025) was performed to predict the functional domains present in four zinc MEPs (*Alternaria alternata* KAH6846781.1, *Pyrenophora seminiperda* RMZ67178.1, *Bipolaris maydis* KAJ5055890.1, and *S. lycopersici* SlMEP1, PQ898935).

### 2.4. Construction of Gene Deletion and Complementary Mutants

The *S. lycopersici* strain SL1216 was used to construct the gene-deleted mutants. The construction of the *SlMEP1* gene-deleted and complementary vectors and subsequent transformation of *S. lycopersici* was performed by a double-joint PCR technique [27] and the polyethylene glycol (PEG)-mediated protoplast transformation [28]. The gene-deleted mutants were identified by PCR using the relevant primers and further analyzed by Southern blotting. The complementary mutants were identified by PCR. All the primers and their sequences used in the study are listed in Table S1.

### 2.5. Pathogenicity Assays and Penetration Determination

The wild-type strain SL1216, the gene-deleted mutant ∆*SlMEP1*, and the complementary strain ∆*SlMEP1*-C were cultured on V8 juice agar plates, and the sporulation was induced as described in Section 2.1, respectively. The spore suspension was inoculated onto the backside of tomato leaves. Six days after the moisturized incubation, the diameter of the lesions on tomato leaves was measured to determine the pathogenicity (reflected by the individual lesion size) of all three strains. To measure the fungal penetration rate, all three strains were placed on V8 agar plates covered with a sterilized cellophane for 36 h before the cellophane was removed from each of the plates along with attached fungal colony. The plates were incubated further for another 48 h, observed for the fungal growth, and photographed. Each treatment was replicated 3 times.

### 2.6. Cell-Death-Inducing Activities and Disease Assays of Heterologously Expressed SlMEP1

The sequence of the *SlMEP1* full-length ORF was sub-cloned into the pCOLD-TF vector and fused with the His-TF (control) tag in the vector to generate the expression vector pCOLD-His-TF-SlMEP1. The successfully constructed vector, as well as the pCOLD-TF vector, were transformed into the competent cells of *Escherichia coli* BL21 (DE3), respectively.

The recombinant His-TF-SlMEP1 protein was expressed after the induction with isopropyl-β-D-thiogalactopyranose (0.5 mM) at 16 °C for 12 h, and purified using Ni^+^ resin (Beyotime Biotech, Shanghai, China). Finally, SDS-PAGE (Solarbio Science & Technology Co., Ltd., Beijing, China) was used to examine the purified proteins His-TF-SlMEP1 and His-TF.

The cell-death-inducing activity of the heterologous expressed recombinant protein His-TF-SlMEP1 was assessed by infiltrating samples (1.0, 2.5, 5.0 µM) into the leaves detached from six-week-old tomato plants. To perform the disease assay, the detached tomato leaves were infiltrated with the proteins for 6 h and then inoculated at the same spots with a drop of 10 µL spore suspension of the *S. lycopersici* SL1216. The treated leaves were placed onto a sheet of sterilized filter paper saturated with distilled water and maintained in an incubator under a 16 h light and 8 h dark schedule at 25 °C. The leaf lesions were measured for length and width, and the photographs were taken within 5 days post inoculation (dpi) of the *S. lycopersici* SL1216. Nine leaves were used for each of treatments with three replicates. The recombinant protein His-TF was used in the same setup as the control.

### 2.7. RNA Extraction and cDNA Synthesis

To test for the transcription profile of *SlMEP1* during the fungal infection on tomatoes, the spore suspension of the *S. lycopersici* SL1216 was transferred into each of 250 mL flasks containing 100 mL PD medium and incubated on a shaker at 160 rpm, 25 °C, for 48 h to obtain newly grown mycelia that were then inoculated onto tomato leaves. The inoculated leaves were subsequently sampled 24, 48, 72, and 96 h post infection of the *S. lycopersici* SL1216. A sample was also taken from the *S. lycopersici* hypha grown in vitro as the control.

To determine whether SlMEP1 inhibits chitinases in tomatoes or not, the qRT-PCR assay was performed to examine the transcriptional level of 5 types of chitinase-encoding genes of tomatoes in the leaves infiltrated with His-TF-SlMEP1 and with the His-TF protein as the negative control. These tested chitinase genes in tomatoes were *Chit3* (FJ849060.1), *Chit4* (XM004248589.3), *Chit9* (XM_004248952.4), *Chit14* (NM001279329.2), and *Chit17* (NM_001247471.1). Their corresponding sequences were aligned in the NCBI to design the qRT-PCR primers specific to detect *SlMEP1* and the chitinases (Table S1).

Total RNAs from each of samples described above were extracted using the TransZol protocol (TransGen Biotech, Beijing, China) following the manufacturer’s instruction. The reverse transcription of each RNA sample was performed using the EasyScript All-in-One First-Strand cDNA Synthesis Super-Mix for qPCR (One-Step gDNA Removal, TransGen Biotech, Beijing, China) for synthesis of first-strand cDNA.

### 2.8. Quantitative Reverse Transcription PCR Analysis of Gene Expressions

Quantitative Reverse Transcription PCR (RT-qPCR) was performed using the TransStart^®^ Top Green qPCR SuperMix (TransGen Biotech, Beijing, China). The amplifications were performed in the 20 μL reactions containing 10 μL of 2 × TransStart^®^ Top Green qPCR SuperMix, 0.4 μL of each primer (10 μM), 2 μL diluted cDNA (1–100 ng), and 7.2 μL of nuclease-free water. The samples were incubated at 94 °C for 5 min as an initial denaturation, followed by 40 amplification cycles at 94 °C for 10 s, 55 °C for 20 s, and 72 °C for 30 s. All the primers used in the study are listed in Table S1.

The relative expression levels of target genes in *S. lycopersici* and the inoculated tomato samples were calculated using the 2^−∆∆CT^ method [29], where the *S. lycopersici β-tubulin* gene (*Slβ-tubulin*) or tomato *Actin* gene (*ToActin*) was used as the internal reference for *SlMEP1* or tomato chitinases genes. These RT-qPCR assays were replicated three times.

## 3. Results

### 3.1. Phylogenetic Analysis and Sequence Alignment of Different Fungal Zinc Metalloproteases

**Figure 1:**
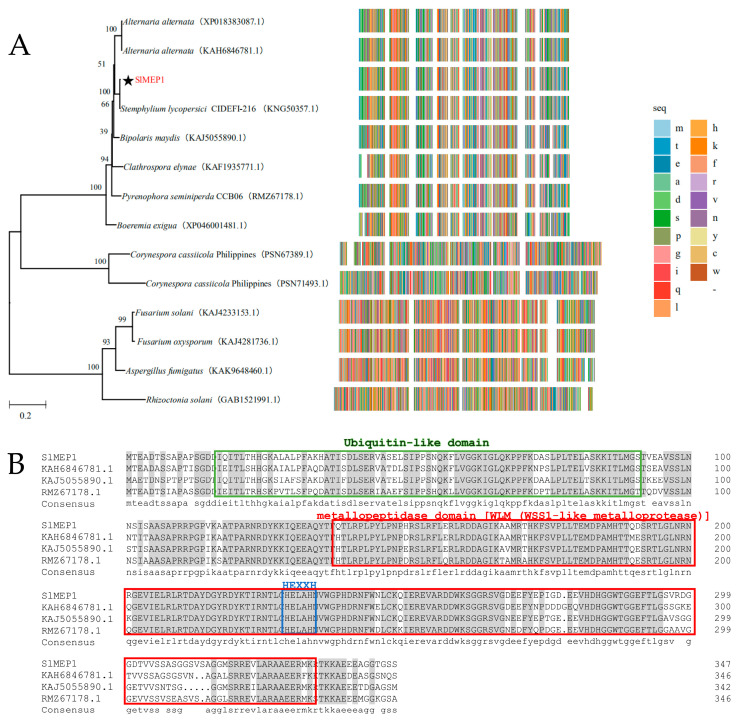
The phylogenetic tree and domain sequence analysis. (**A**) The phylogenetic tree constructed by the full-length sequence alignment of homologous metalloproteases of *S. lycopersici* and other fungi. (**B**) The sequence alignment of the metalloproteases. The green and red boxes represent the ubiquitin-like domain and WLM (WSS1-like metalloprotease) domain predicted online using Pfam (http://pfam.xfam.org/, accessed on 10 February 2025), respectively. The blue box is the HEXXH site specific to the zinc metalloprotease. The star in (**A**) indicates SlMEP1 of *S. lycopersici*.

The *SlMEP1* gene (PQ898935) that encodes a zinc MEP protein with 347 amino acids was identified by the RNA sequencing from the *S. lycopersici* strain SL1216, and the transcriptional dynamics of SL1216 genes indicated an upregulated trend during the early stage of the infection. Multiple comparisons of the sequences in Figure 1A showed that there were genes that share a high homology to the SlMEP1 of *S. lycopersici*, *Alternaria alternata*, *Bipolaris maydis*, and *Pyrenophora* sp. A homology alignment (http://pfam.xfam.org/, accessed on 10 February 2025) indicated a ubiquitin-like domain (17–91 AA), a WLM (WSS1-like MEP) domain (138–332 AA), and a characteristic HEXXH-active site for MRPs in SlMEP1, which was similar to those of other fungi (*Alternaria alternata* KAH6846781.1, *Pyrenophora seminiperda* RMZ67178.1, and *Bipolaris maydis* KAJ5055890.1) (Figure 1B). To understand the evolutionary relationship among SlMEP1 and MEPs of other fungi, a phylogenetic tree based on the amino acid sequences was constructed using the neighbor-joining phylogeny method [30], which showed that SlMEP1 was clustered into the same clade (100%) as the zinc MEPs of the *S. lycopersici* strain CIDEFI-216 (KNG50357.1) and highly close to other two zinc MEP-like proteinases (86.78% and 86.49%) of *Alternaria alternata* (KAH6846781.1, XP018383087.1) (Figure 1A). Both homologous alignment and phylogenetic analysis indicated that the zinc MEPs of *S. lycopersici* is evolutionarily conserved.

### 3.2. Transcriptional Profiling of SlMEP1 During Fungal Pathogenesis of S. lycopersici to Tomato

**Figure 2:**
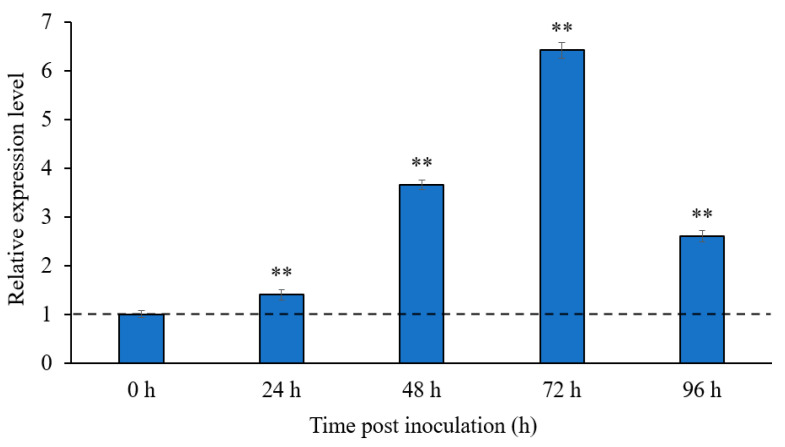
Transcriptional levels of the SlMEP1 gene of *S. lycopersici* during the fungal invasion to tomato leaves. Error bars were calculated based on three replicates, and the asterisks ** indicate a significant difference (*t*-test; *p* < 0.01) between the transcriptional level of the *S. lycopersici*-inoculated samples and in vitro *S. lycopersici* hypha.

The transcriptional *SlMEP1* profile of the *S. lycopersici* strain SL1216 was generated during the fungal invasion to tomato leaves at different sampling times: 24 h, 48 h, 72 h, and 96 h post inoculation (hpi) and was compared with the profile of *S. lycopersici* hypha in vitro (0 hpi). Quantitative RT-PCR (RT-qPCR) analyses indicated that in vitro *SlMEP1* hypha displayed the lowest transcriptional level, while the transcriptional levels of SL1216-*SlMEP1* during the fungal invasion were significantly upregulated and reached the peaks (3.65~6.42-fold) at 48 and 72 hpi (Figure 2), suggesting an important role that SlMEP1-related MEPs played during the early pathogenesis of the tomato-invading fungus.

### 3.3. Reduced Pathogenicity of S. lycopersici Due to Deletion of SlMEP1

The *SlMEP1*-deleted mutant ∆*SlMEP1* and complementary strain ∆*SlMEP1*-C of the *S. lycopersici* SL1216 were constructed by the protoplast transformation and were validated by PCR. ∆*SlMEP1* was further confirmed by Southern blotting (Figure S1).

**Figure 3:**
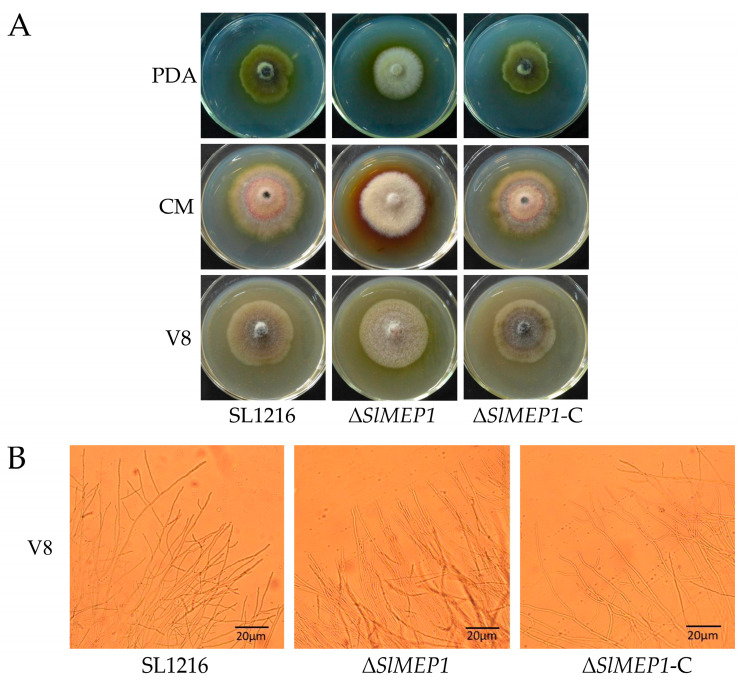
The impact of the *SlMEP1* deletion on the hyphal growth of *S. lycopersici*. (**A**) The colony of the wild-type strain SL1216, mutant strain Δ*SlMEP1*, and complemented strain Δ*SlMEP1*-C cultured on the potato dextrose agar (PDA), corn meal agar (CMA), and V8 juice agar (V8) plate at 25 °C for 5 days. (**B**) Hyphal branches of the wild-type strain SL1216, mutant strain Δ*SlMEP1*, and complemented strain Δ*SlMEP1*-C at the edge of V8-coated slides after 24 h (bar = 20 μm).

To study the effect of *SlMEP1* on the fungal growth and nutrient utilization, the strains of SL1216, Δ*SlMEP1*, and Δ*SlMEP1*-C were cultured separately on the PDA, CMA, and V8 plates (Figure 3A). Five days after incubation, the ∆*SlMEP1* strain showed significant differences in the pigment production and mycelial density compared to those of the SL1216 and ∆*SlMEP1*-C strains on all three medium plates, except that of the mycelial growth rate. The microscopic observation of mycelia grown on the V8 plates revealed that ∆*SlMEP1* hypha were unable to branch as normally as those of the SL1216 or ∆*SlMEP1*-C strains. Some forked branches at the tip of ∆*SlMEP1* hypha on the V8 plates were noticed (Figure 3B).

**Figure 4:**
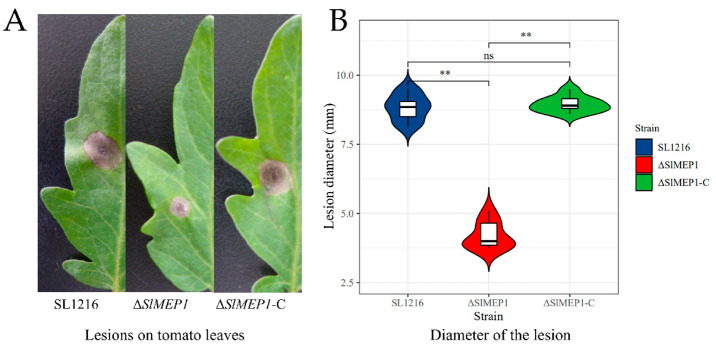
Comparison of lesion sizes induced by three *S. lycopersici* strains, ∆*SlMEP1*, SL1216, and ∆*SlMEP1*-C. (**A**) Lesion size and appearance on tomato leaves inoculated with three different strains for 6 days. (**B**) Statistical comparison of lesion diameters (mm) on tomato leaves after inoculation. Asterisks ** indicate a significant difference (*p* < 0.01, *t*-test, n = 9) between each of three strains (ns = not significant).

To decipher the relationship between *SlMEP1* and the pathogenicity of *S. lycopersici*, each spore suspension of three *S. lycopersici* strains was inoculated onto tomato leaves to determine their pathogenicity by measuring the lesion size. After being cultured at 25 °C for 6 days, the ∆*SlMEP1* mutant induced the smallest necrotic lesions, while the *S. lycopersici* wild-type and ∆*SlMEP1*-C strain produced larger lesions (Figure 4). Therefore, the pathogenicity of the ∆*SlMEP1* mutant was significantly reduced.

### 3.4. Impaired Hyphal Penetration of S. lycopersici Due to SlMEP1 Deletion

**Figure 5:**
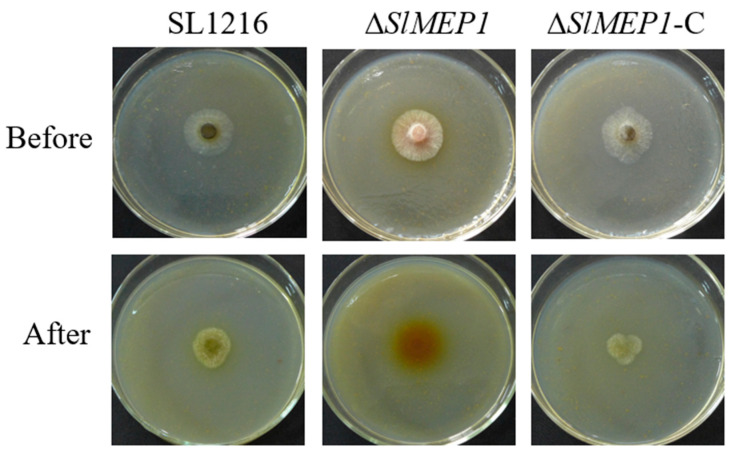
Comparison of three *S. lycopersici* strains, ∆*SlMEP1*, SL1216, and ∆*SlMEP1*-C, grown on the cellophane-membrane-covered V8 agar plates for 36 h first (Before) and then for another 48 h after the removal of the membrane (After), showing that the *SlMEP1*-deleted ∆*SlMEP1* strain was unable to penetrate the cellophane membrane.

The strains of Δ*SlMEP1*, SL1216, and the Δ*SlMEP1*-C were grown on the cellophane-membrane-covered V8 agar plates for 36 h first and then for another 48 h after the membrane was removed from the plates. Unlike SL1216 and Δ*SlMEP1*-C, the Δ*SlMEP1* strain was unable to penetrate the cellophane membrane (Figure 5), indicating that the *SlMEP1* gene and its expression were needed for *S. lycopersici* to penetrate the cellophane membrane.

### 3.5. SlMEP1 Induced Cell Collapse in Inoculated Tomato Leaves

**Figure 6:**
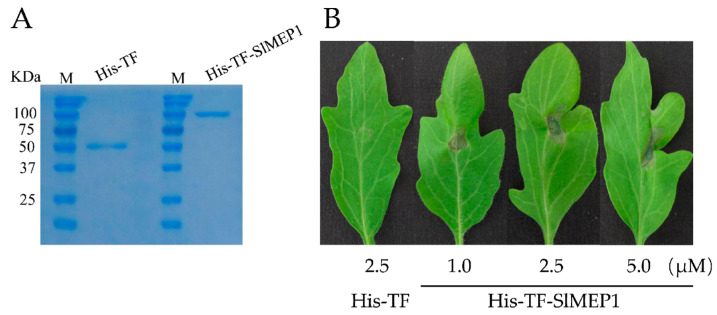
Expression of necrotic lesions on tomato leaves induced by the SlMEP1 protein. (**A**) SDS-PAGE confirmation of the heterologous expression of purified His-TF-SlMEP1 and His-TF proteins. (**B**) The cell collapses of lesions on leaves of tomato cultivar “Jinpeng No. 1” induced by the His-TF-SlMEP1 and His-TFS lMEP1 proteins 3 days after inoculation.

To further confirm the role that the SlMEP1 protein plays in viral pathogenicity, an expressing vector of the pHis-TF-SlMEP1 recombinant protein was constructed to highly express the His-TF-SlMEP1 recombinant protein in *Escherichia coli* BL21 (DE3), which was further quantified by sodium dodecyl sulphate-polyacrylamide gel electrophoresis (SDS-PAGE) after its purification (Figure 6A).

Three days after inoculating the purified His-TF-SlMEP1 protein into leaves of the susceptible tomato cultivar “Jinpeng No. 1” at a concentration of 1.0, 2.5, or 5.0 µM, respectively, the His-TF-SlMEP1-inoculated spots clearly expressed a sign of cell collapse/necrosis, while the His-TF-infiltrated leaves as the control (CK) did not (Figure 6B). The necrotic size of the His-TF-SlMEP1-inoculated spots enlarged along with the increase in the His-TF-SlMEP1 concentration.

### 3.6. Enhanced Pathogenicity of S. lycopersici Due to Added SlMEP1 Protein

**Figure 7:**
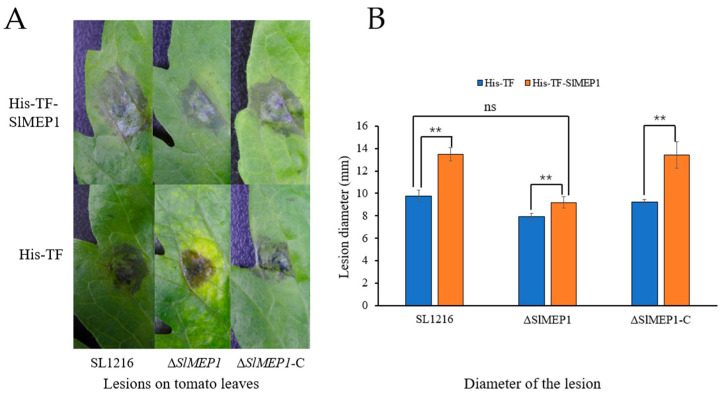
Induced cell collapse/necrosis in SlMEP1-infiltrated tomato leaves. (**A**) Lesions showing cell collapses on tomato leaves inoculated with His-TF-SlMEP1 and His-TF proteins in response to inoculation of the Δ*SlMEP1*, SL1216, and Δ*SlMEP1*-C strains. (**B**) Diameter of lesions induced by each combination of inoculated proteins and *S. lycopersici* strains on tomato leaves. Asterisks ** indicate a significant difference between the His-TF-SlMEP1 and His-TF (CK) treatment (*t*-test; *p* < 0.01, n = 9, and ns = not significant).

To further determine the virulent role that SlMEP1 plays, the purified recombinant His-TF-SlMEP1 and His-TF proteins were first injected into tomato leaves separately and then each spore suspension of the Δ*SlMEP1*, SL1216, and Δ*SlMEP1*-C strains were inoculated onto the protein-infiltrated leaves. The disease progress virulence was assessed by the lesion size including the water-soaked area 5 days after the second inoculations were completed. Lesions on the His-TF-SlMEP1-infiltrated leaves inoculated with each of three different *S. lycopersici* strains enlarged faster and grew bigger in comparison with those on the His-TF-infiltrated leaves inoculated with the same strain as the controls (Figure 7A). The statistical analysis further validated the significant difference between all different His-TF-SlMEP1 and the His-TF treatments across all three *S. lycopersici* strains, but no significant difference was found between the His-TF-SlMEP1 plus Δ*SlMEP1*, His-TF plus SL1216, or His-TF plus Δ*SlMEP1*-C treatments (Figure 7B). The comparisons of lesion sizes indicated that the SlMEP1 protein had compensated the reduced virulence due to deletion of the *SlMEP1* gene in *S. lycopersici* as the virulent factor affecting its pathogenicity.

### 3.7. The Chitinase Gene Expression in Tomato Affected by the SlMEP1 Protein

**Figure 8:**
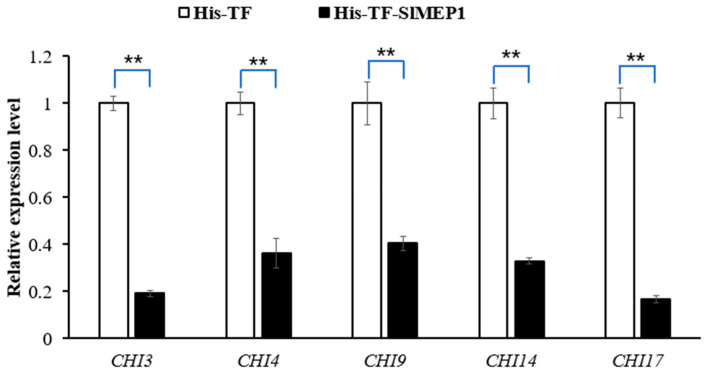
Inhibited expression of chitinases due to the SlMEP1 protein in the His-TF-SlMEP1 or His-TF-infiltrated tomato leaves at a concentration of 2.5 µM two days after inoculation. The tomato actin gene was used as an internal control to normalize the data. Error bars represent the standard error. Asterisks ** indicate significant difference between the His-TF-SlMEP1 and His-TF (CK) treatments (*t*-test; *p* < 0.01 and n = 3).

The RT-qPCR detection of chitinase *Chit3*, *Chit4*, *Chit9*, *Chit14*, and *Chit17* in the His-TF-SlMEP1 or in the His-TF-inoculated tomato leaves indicated that the transcript levels of all of these five chitinase-encoding genes were significantly reduced in the tomato leaves infiltrated with His-TF-SlMEP1, compared with the control leaves infiltrated with His-TF (Figure 8), suggesting that the SlMEP1 protein inhibited the expression of all chitinases tested.

## 4. Discussion

As a necrotrophic fungal pathogen [31,32] of many plant hosts, *S. lycopersici* is economically important due to its potential capability of causing devastating diseases to many “cash” vegetable crops, such as tomatoes, peppers [4], eggplants [33], lettuces [34], physalises (*Physalis alkekengi*) [35], asparaguses [36], and watermelons [37]. Among its hosts, tomatoes often suffer the most [10]. In recent years, several new hosts, such as spinaches (*Spinacia oleracea*) [38] and *Salvia splendens* [39], have been added to the host list. However, the control of diseases caused by *S. lycopersici* solely relies on use of fungicides, and more advanced, safe, and environmentally friendly strategies for disease management are sought to face the disease challenge. Therefore, understanding the molecular mechanisms of pathogenesis of *S. lycopersici* is the first and foremost step.

The research work on the genome sequencing assembly and annotation [40,41], transcriptome analysis [20], and prediction of pathogenesis-related effectors of *S. lycopersici* [42,43] has laid an important foundation for deciphering the molecular mechanism of pathogenesis of *S. lycopersici*. The necrosis- and ethylene-inducing peptide 1-like protein (NLP) gene is cloned from *S. lycopersici* and its targeted gene replacement is carried out. Subsequent studies on the role of NLP in pathogenicity, asexual reproduction, growth and development, and environmental stress response have revealed that NLP is a virulence factor of *S. lycopersici* [44]. However, there are few reports on the functional studies of other genes pertaining to *S. lycopersici* pathogenicity. In this study, we have identified a zinc metalloprotease gene *SlMEP1* from the *S. lycopersici* genome and transcriptome data. This first reported investigation focused on providing scientific evidences to validate its functional role in the fungal pathogenicity by investigating the much-needed zinc metalloprotease gene in *S. lycopersici*.

Metalloproteases characterized in pathogenic fungi are largely found to be involved in their pathogenicity. With many other phytopathogenic fungi, there is evidence from various research that several metalloproteases play an important role in virulence [11,12,13,14]. Lv et al. [45] found that two metalloproteinases with the M35 family from *Verticillium dahliae*, VdM35-1 and VdASPF2, are involved in the reduced sporulation, slow growth of hyphal branches, and distorted shape of spores through their gene deletions. In addition, the pathogenicity of their gene-deleted mutants is significantly reduced [45]. In *Rhizoctonia cerealis*, whole-genome metalloproteases are identified and characterized, among which the function of metalloproteases in the M35, M36, and M43 family; RcMEP2, RcFL1, and RcMEP1 are studied intensively. The transcript levels of these three MEPs significantly increased during *R. cerealis* infection of wheat, causing wheat cell collapses by promoting pathogen virulence and repressing the expression of wheat genes encoding chitinases, according to the gene-function analysis [16,17,18]. In our study, the *SlMEP1* gene in *S. lycopersici* was greatly expressed at a high level during the fungal invasion of tomatoes (from 24 to 96 hpi). The *SlMEP1*-deleted mutants also exhibited a decelerated mycelial growth and reduced mycelial penetration and pathogenicity. Treatments of tomato leaves with heterologously expressed MEP proteins showed that SlMEP1 was able to induce necroses and cell collapses, thus enhancing the pathogenicity of *S. lycopersici* in the inoculated tomato leaves. These findings are generally consistent with those reported previously, which confirms that SlMEP1 is a pathogenicity-required or -associated factor during the process of *S. lycopersici* infection in tomato. According to studies on the functional role of MEPs in the M35 and M36 families in *F. graminearum*, FgM35 and FgFly1 are involved in the fungal reproduction and pathogenicity by interacting with TaZnBP and CAMTA proteins, respectively, in wheat to regulate the pathogenicity of *F. graminearum* [19], which provides us a new insight for our further study on *S. lycopersici* MEPs regarding pathogen–host interactions.

Plants have evolved to adapt or resist fungal infections by utilizing specific enzymes such as chitinases capable of degrading fungal cell walls as a fundamental component of plant defensive responses. The release of chitin monomers is demonstrated to facilitate the elimination of pathogenic fungi in plants, which is widely considered to be a defensive mechanism that is evolutionarily conserved [17]. In response, phytopathogenic fungi have evolved various proteases, including MEPs and serine proteases, to degrade chitinases and, thereby, to repress the chitin-mediated defense responses of plants [14,46,47]. For instance, the *FoMEP1*- and *FoSep1*-deleted mutants of *F. oxysporum* are unable to cleave chitinases and are less virulent in inoculated tomato plants, demonstrating a critical role of plant chitinase cleavage pertaining to fungal virulence [25]. The M43 domain-containing MEPs such as RcMEP1 and the M35 family MEPs in *R. cerealis* are proven to decrease the expression of chitinases in wheat [14,16,17,22,24]. While it has remained unknown how *S. lycopersici* counteracts with the host chitinases, the present findings demonstrate that the heterologous expressed SlMEP1 reduces the expression of five distinct types of chitinases in tomato, thereby corroborating with the conclusions reported before.

Many recent reports reveal that a reduction in pathogenicity caused by the deletion of functional protein genes is sometimes accompanied by a reduced penetrating ability of mycelia. In *Verticillium dahlia*, the knockout experiments on deleting the NADPH oxidase (Nox) encoding gene (*NOXA*), trehalase encoding gene (*VdPT1*), and hypothetical protein encoding gene *VDAG*_07742 confirm that the gene-deleted mutants exhibit several notable changes in their characteristics, including the slowed mycelial growth, reduced conidial production, decreased mycelial penetration, and lessened virulence against plant hosts [48,49,50]. In contrast, the deletion of the *VdxyL3* gene leads to an increased penetrating capacity and the enhanced pathogenicity of *V. dahlia* [51]. The SUMOylation represents a pivotal post-translational modification, wherein the small ubiquitin-related modifier (SUMO) proteins exert a regulatory influencer during the pivotal biological processes, including the pathogenesis of phytopathogenic fungi. A disruption of key SUMOylation pathway genes significantly reduces the pathogenicity, impairs the penetration ability, and attenuates the invasive growth capacity of *Fusarium oxysporum* f. sp. *Niveum* [52]. In the present study, we confirmed that *SlMEP1* of *S. lycopersici* is indispensable in penetrating the cellophane membrane, as the *SlMEP1*-deleted mutants were unable to break through the cellophane membrane in 2 days after inoculation. Meanwhile, the *SlMEP1*-deleted mutants were also less pathogenic than the wild-type strain, suggesting a positive correlation between the mycelial penetration and fungal pathogenicity.

In summary, these results suggest that the zinc metalloprotease SlMEP1 acts as an important pathogenicity-regulating factor during the infection of *S*. *lycopersici* in tomatoes, and its presence also ensures the mycelial penetration, helps *S. lycopersici* to break through host cuticle and cell walls to colonize the host interior tissues, and evades host defensive barriers by inhibiting the expression of host gibberellinase gene. Moreover, the protein also facilitates the nutrient uptake and expansion of *S*. *lycopersici* within the host cells by causing tissue necrosis. In this study, *SlMEP1* has been proven to be a candidate gene for improving tomato resistance against *S. lycopersici* through silencing a host-pathogenicity-inducing gene.

## 5. Conclusions

Our study has deciphered the functions of a metalloprotease SlMEP1 of *S. lycopersici*, indicating that the SlMEP1 protein is one of the MEPs that induces the cell collapses and necroses in host plant leaves, manipulates fungal penetrating ability, inhibits the expression of chitinase-encoding genes, and facilitates the virulence against tomato plants during the pathogenesis of *S. lycopersici*. The findings derived from this study should shed a light on developing novel strategies for disease prevention and management following the initial infection of *S. lycopersici*, thereby contributing to the implementation of integrated disease management practices in the control of tomato gray leaf spot.

## Data Availability

The original contributions presented in this study are included in the article/Supplementary Materials. Further inquiries can be directed to the corresponding authors.

## Supplementary Materials

The following supporting information can be downloaded at: https://www.mdpi.com/article/10.3390/jof11050330/s1. Figure S1: Confirmation of the *SlMEP1* gene knockout mutant and its complemented *S. lycopersici* strains; Table S1: Primers used in this study.
