# Supplementary material for: Zinc Metalloprotease SlMEP1: An Essential Factor Required for Fungal Virulence in Stemphylium lycopersici
Source: J Fungi (Basel). 2025 Apr 22;11(5):330. doi: 10.3390/jof11050330 (PMC12112490; doi:10.3390/jof11050330)
Supplement: Supplementary file 1 [file jof-11-00330-s001.zip › jof-3528010-supplementary.pdf]

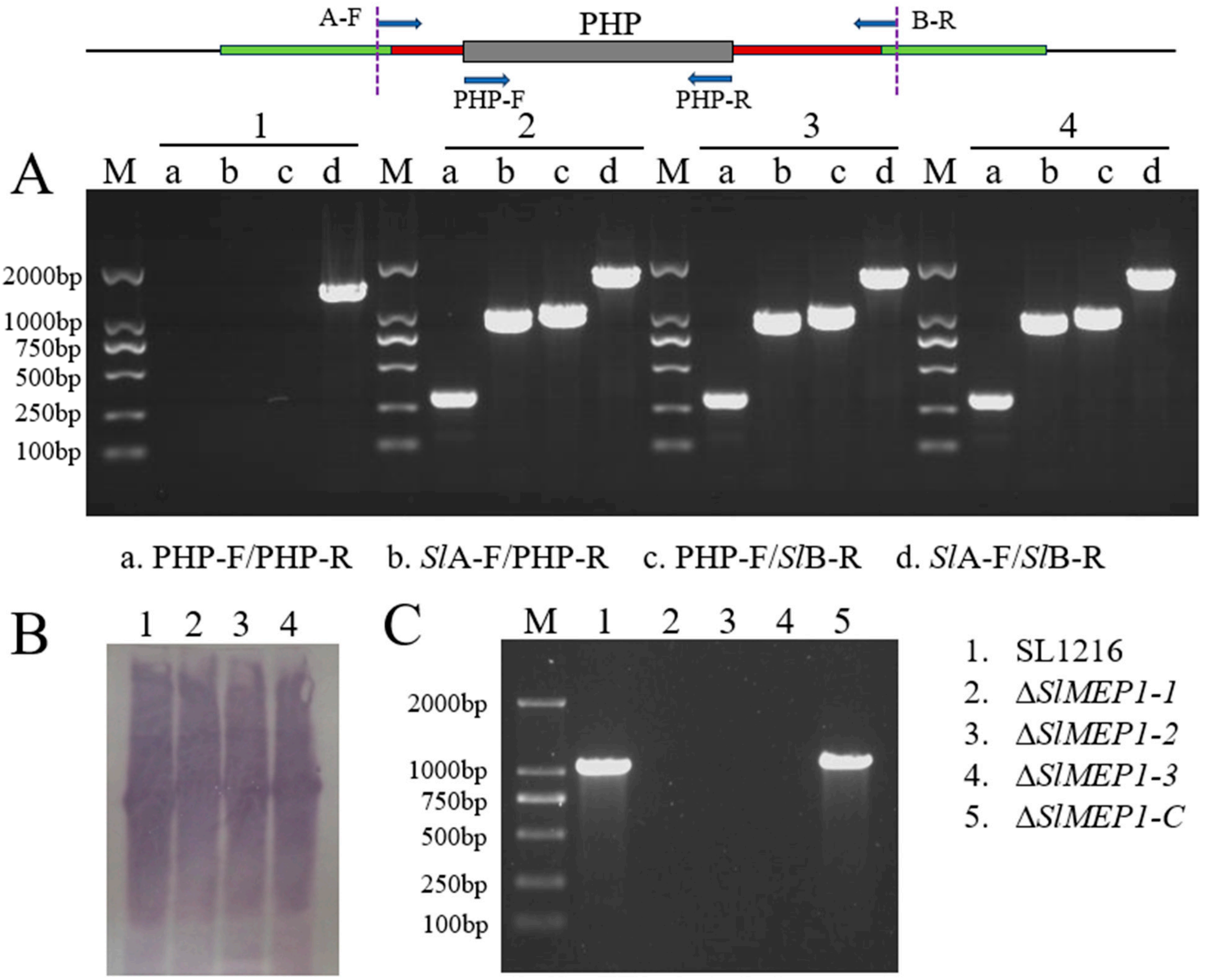

Figure S1. Confirmation of the *SlMEP1* gene knockout mutant and its complemented *S. lycopersici* strains. (**A**): PCR results showing the *SlMEP1* gene knockout mutant. (**B**): Southern blot analysis to confirm the *SlMEP1* gene knockout mutant. (**C**): PCR results showing the detection of the *SlMEP1* gene in the knockout mutant Δ*SlMEP1* and the complementary strain Δ*SlMEP1*-C.

Table S1. Primers and their sequences in this study.

Primer name	Sequence (5'–3')	Usage
SlMEP1-F	ATGACCGAAGCAGACACATC	PCR for *SlMEP1* cDNA amplification/PCR for *ΔSlMEP1*-C detection
SlMEP1-R	TTACGAACTACCCGTACCAC	
PHP-F	GATGTAGGAGGGCGTGGATATG	PCR for *ΔSlMEP1* mutant detection
PHP-R	GTATTGACCGATTCCTTGCGG	
SlA-F	GGGCTGCCGATGAATAGTAA	
SlB-R	GGGTGAAAACCGAAAAGTCA	
Q*Slβ-tubulin*-F	CGAGCGTATGAACGTCTACTT	RT-qPCR for *S. lycopersici β-tubulin* transcript
Q*Slβ-tubulin*-R	GCACGAACTTGTTGTTGGAG	
Q*ToActin*-F	GGAAAAGCTTGCCTATGTGG	RT-qPCR for tomato *Actin* transcript
Q*ToActin*-R	CCTGCAGCTTCCATACCAAT	
Q*ToChit3*-F	GTTGTGGATGACAGAACAGGA	RT-qPCR for tomato *chitinase3* transcript
Q*ToChit3*-R	ACCGTACCCTGGAACTCTATTA	
Q*ToChit4*-F	CTCAAACCTCCCACGAAACTA	RT-qPCR for tomato *chitinase4* transcript
Q*ToChit4*-R	CTGGGCTACCTTGTTCTCTAAG	
Q*ToChit9*-F	GAGGCCCAATCCAAATTTCAC	RT-qPCR for tomato *chitinase9* transcript
Q*ToChit9*-R	CTGGGTCTGTGGCTACTAAATC	
Q*ToChit14*-F	GTCCCTCTGTTGCTGATTCT	RT-qPCR for tomato *chitinase14* transcript
Q*ToChit14*-R	GAACCGCTGTTACATTCCATTC	
Q*ToChit17*-F	TGACCGCACAAGGCAATAA	RT-qPCR for tomato *chitinase17* transcript
Q*ToChit17*-R	CCGTAGCCTGGTTGTCTATTT	
